# The Prevalence of Congenitally Missing Permanent Teeth in a Sample of Orthodontic and Non-Orthodontic Caucasian Patients

**DOI:** 10.3390/healthcare12050541

**Published:** 2024-02-24

**Authors:** Nefeli Katanaki, Miltiadis A. Makrygiannakis, Eleftherios G. Kaklamanos

**Affiliations:** 1 Private Practice in Mytilini, 81100 Lesvos, Greece; nk181453@students.euc.ac.cy; 2 School of Dentistry, National and Kapodistrian University of Athens, 11527 Athens, Greece; 3 School of Dentistry, Aristotle University of Thessaloniki, 54124 Thessaloniki, Greece; ekaklam@dent.auth.gr; 4 School of Dentistry, European University Cyprus, Nicosia 2404, Cyprus; 5 Hamdan Bin Mohammed College of Dental Medicine, Mohammed Bin Rashid University of Medicine and Health Sciences, Dubai P.O. Box 505055, United Arab Emirates

**Keywords:** hypodontia, congenitally missing teeth, orthodontics

## Abstract

Background: Hypodontia represents a notable clinical and public health concern. Objective: To assess the prevalence of congenitally missing permanent teeth in a sample of orthodontic/dental patients of Caucasian origin originating from the Greek island of Lesvos. Materials and Methods: Panoramic X-rays from 621 children and adolescents, aged 9 to 16 years (average age 12.5 years), 521 seeking orthodontic care (orthodontic group) and 100 seeking dental care (non-orthodontic group) were examined to identify congenitally missing permanent teeth. Results: The orthodontic group exhibited a 5.5% prevalence of congenitally missing permanent teeth (2.8% females; 2.7% males), while the non-orthodontic group showed a prevalence of 4% (3% females; 1% males). The descending order of prevalence for missing tooth types was as follows: lower second premolars, upper laterals, lower central incisors, lower canines, upper second premolars, and lower second molars. Among orthodontic patients with missing teeth, 62% presented with an Angle’s Class II malocclusion. Hypodontia was most frequently observed in the mandible. No statistically significant differences were observed between the orthodontic and non-orthodontic groups in terms of the percentage of children and types of congenitally missing teeth. Conclusions: Congenitally missing teeth were observed in about 4–5% of the studied population with a female predilection. The lower second premolar was the most commonly absent tooth, followed by the maxillary lateral incisors. An Angle’s Class II malocclusion was present in the majority of orthodontic patients with hypodontia, mostly in the mandible.

## 1. Introduction

The congenital absence of teeth or dental agenesis arises from a failure during the initiation stage of tooth development within the dental lamina [1,2,3]. Research suggests that dental agenesis more commonly impacts the permanent than the primary dentition [4], posing significant challenges from both clinical and public health aspects [1,2]. The severity of the condition varies from hypodontia (fewer than six permanent teeth missing, excluding third molars) to oligodontia (six or more teeth missing) and anodontia (complete absence of teeth) [3,5,6,7].

The congenital absence of permanent teeth, excluding third molars, exhibits a prevalence of 3–10% or approximately one in every 10–12 individuals, with a 3:2 female-to-male predilection [1,2,8]. Typically, variations in tooth number affect the most distal tooth of a type, such as third molars, second premolars, and lateral incisors [9,10]. Noteworthy is the significantly lower incidence of congenitally missing teeth among African Americans [1,2].

The origin of non-syndromic, familial congenitally missing permanent teeth remains unknown and is considered multifactorial [11]. With over 200 genes involved, genetics play a pivotal role in the causation of tooth agenesis. Genetic mutations are frequently linked to hypodontia, likewise, AXIN2 gene mutations are inherited as autosomal dominant disorders [11] and are usually associated with congenitally missing posterior teeth [12]. Other associations with genes have been documented, including PAX9, MSX1 [13,14,15,16,17], LTBP3, and EDA [18]. Among these genes, the PAX9 gene mutation follows an autosomal dominant pattern, leading to the absence of teeth, with molars being the ones most usually affected. Inheritance can also exhibit recessive or X-linked patterns, displaying notable variability in both expression and penetrance [12].

Meanwhile, more than 50 syndromes and conditions exhibit hypodontia as a major feature [11]. These include ectodermal dysplasia, Down syndrome, chondroectodermal dysplasia, Rieger syndrome, craniosynostosis syndrome, incontinentia pigmenti, oro-facial-digital syndrome, Williams’ syndrome, and dentoalveolar clefting with disruption of the dental lamina at that site. In the case of multiple dental anomalies, genetic counseling in combination with radiographic evidence is recommended [19,20]. Given the genetic correlation observed between the congenital absence of permanent teeth and adenomatous polyposis as well as colorectal cancer, it is strongly recommended to conduct a comprehensive assessment of the family’s medical history [12].

Dental characteristics and occlusal features commonly associated with the absence of maxillary lateral incisors include delayed dental development, microdontia of the contralateral maxillary lateral incisor, palatal displacement of maxillary canines, and distal angulation of mandibular second premolars. It is hypothesized that various distinctive phenotypic expressions may be linked to genetic mutations [19,20]. Additionally, another potential factor leading to unilateral or bilateral tooth agenesis could be exposure to radiation in the maxillofacial area during early childhood. This is attributed to the heightened sensitivity of developing permanent teeth to radiation, wherein even a low dosage can impact tooth buds or, if partial calcification has occurred, it may result in observable root stunting [21,22].

The objective of this study was to investigate the prevalence of congenitally missing permanent teeth in a sample of dental patients of Caucasian ancestry originating from the Greek island of Lesvos. Located in the northeastern Aegean Sea, Lesvos is the third largest among the Greek islands and ranks eighth in size among all the islands in the Mediterranean. Its history of human habitation dates back to at least 3000 BC and, at present, Lesvos is home to a population of approximately 85,000 residents. Inhabited since the Paleolithic period, this important Greek island has undergone various historical epochs, including the Ancient Greek, Byzantine, Ottoman, and modern Greek eras, much like the majority of the present-day Greek territories. Over the centuries, it has become evident that individuals from diverse genetic backgrounds have lived on the island, potentially leaving their mark throughout different time periods. Given the fact that, apart from its continental territories, Greece has a significant number of islands, the authors of the present paper thought that the investigation of congenitally missing teeth on the island of Lesvos would be an interesting starting point.

## 2. Materials and Methods

### 2.1. Ethical Approval

In order to carry out this study, we followed the relevant guidelines and regulations. Approval for the project was obtained from the relevant entity of the private practice in Mytilini, Lesvos (the Ethical Committee of Dr. M. Katanakis’ Clinic), where the diagnostic records of the patients originated (#4 on 12 October 2022). Prior consent had also been obtained from the patients. Patient files including panoramic radiographs, dental casts, and any available specific periapical radiographs were evaluated as the only resources of diagnostic information. A tooth was registered as congenitally missing when no trace could be found on the radiograph and treatment records could confirm that it was not extracted.

The investigation was conducted anonymously, in a private practice on Lesvos island, based on the archive material of pre-existing records and no intervention took place. The study involved a convenience sample of 521 children (258 males and 363 females) attending orthodontic clinics and 100 attending general/pediatric practices, drawn from larger groups of 590 and 119 children, respectively. The orthodontic sample could display a greater chance of presenting hypodontia because patients with congenitally missing teeth would be more likely to seek orthodontic treatment for the management of their missing teeth [23]. Therefore, one benefit of comparing an orthodontic sample to a non-orthodontic sample is the ability to mitigate selection bias. Having two distinct groups enables the assessment of the potential differences between them and helps to avoid overestimating the prevalence of hypodontia.

The inclusion criteria involved children and adolescents aged 9 to 16 years [24], with both parents being native Greeks from the island of Lesvos, and the availability of high-quality and clear panoramic X-rays. Offsprings of parents not of Greek origin or from other Greek regions were excluded. Children with extractions of permanent teeth due to caries, trauma, or orthodontic treatment, as well as individuals with syndromes and conditions linked to hypodontia (e.g., ectodermal dysplasia, cleft lip and palate, etc.), were also not considered.

The reason why children of nine years of age or older were recruited for this research is as follows: the calcification of the tooth’s crown is usually completed at six years of age [25] but, in some individuals, there can be a delayed development of premolars [26]. Hence, we cannot be sure if they are missing or not below the age of 9, and especially in males [27]. Moreover, it has been proven that the prevalence of missing teeth was higher in 7-year-old children compared to the same children at 9 years of age [28].

### 2.2. Study Details

The study conducted was a retrospective cross-sectional study and involved a convenience sample of 521 children attending orthodontic clinics and 100 attending general/pediatric practices (258 males and 363 females), drawn from larger groups of 590 and 119 children, respectively.

The prevalence of congenitally missing permanent teeth, excluding third molars, was assessed. Additionally, orthodontic patient records were examined to eliminate the possibility of permanent teeth extraction due to caries or orthodontic reasons and to obtain Angle’s classification of malocclusion. All radiographs were evaluated on the dental X-ray viewer by two authors (N.K. and E.G.K.) whose education and university studies enabled them to assess panoramic radiographs.

Descriptive statistics were computed and, to test the differences in the distribution of congenitally absence teeth, a series of chi square tests were conducted. All statistical analyses were performed with the IBM SPSS v.29.0 enhanced with the module Exact Tests [29]. The significance level in all hypotheses and testing procedures was predetermined at a = 0.05 (*p* ≤ 0.05).

## 3. Results

The mean age of the 621 children included in the study group was 12.5 years. The prevalence of congenitally missing permanent teeth was 5.5% in the orthodontic (14 males and 15 females) and 4% in the non-orthodontic group (1 male and 3 females) (Table 1).

Most congenitally missing teeth in the orthodontic sample were found in the mandible with no difference between genders (*p* = 0.47, Chi-square test). The most commonly missing tooth types were the lower second premolars (n = 20), followed by upper laterals, lower central incisors (n = 7), lower canines, upper second premolars, and lower second molars (n = 2) (Table 2). In the non-orthodontic sample children, three upper laterals and two lower second premolars were identified as congenitally absent (Table 2). No statistically significant differences were observed between the two groups concerning the percentage of children with congenitally missing teeth (*p* = 0.53, Chi-square test), gender (*p* = 0.60, Chi-square test), tooth type (*p* = 0.68, Chi-square test), and quadrant (*p* = 0.64, Chi-square test).

In the orthodontic group children, most individuals with congenitally missing teeth exhibited an Angle’s Class II malocclusion (62%), followed by Class I and Class III (Table 3). No statistically significant differences were observed between the different Angle’s Classes concerning the number of males and females with congenitally missing teeth (*p* = 0.24, Chi-square test).

In Angle’s Class I malocclusion children, the lower second premolars were observed to be congenitally missing more frequently and mostly in females, followed by upper laterals, upper second premolars, and canines. Similar trends were observed in children with an Angle’s Class II malocclusion, whereas in the male patient with an Angle’s Class III malocclusion, an upper right lateral incisor was found missing (Table 4). No statistically significant differences were observed between the children with Angle’s Class I and II malocclusions concerning the type of missing tooth (*p* = 0.68, Chi-square test) and quadrant (*p* = 0.55, Chi-square test). Most teeth congenitally missing were found in the mandible.

## 4. Discussion

The absence of permanent teeth is typically suspected in early childhood, with the initial sign often being a submerged primary tooth or its prolonged retention [1,2]. Nevertheless, the absence must be corroborated using evidence from radiographic examination, usually panoramic or periapical X-rays.

On the present study, we excluded individuals with syndromes and conditions that are linked to hypodontia to avoid overestimation of the results and maintain a representative population sample. Nevertheless, a convenience sample with criteria was used. Also, the sample consisted of two subgroups: an orthodontic group and a non-orthodontic group. A greater prevalence of congenitally missing teeth in the orthodontic group could be anticipated to be observed, as individuals with congenitally missing teeth may be more likely to seek orthodontic treatment to address their tooth absence [23]. Consequently, comparing an orthodontic sample to a non-orthodontic sample becomes advantageous for mitigating selection bias. This approach, involving two distinct groups, facilitates the evaluation of potential disparities and prevents an overestimation of hypodontia prevalence.

Overall, our study was conducted in accordance with a protocol that was formed at the beginning of this investigation. The researchers were trained and calibrated to be able to detect congenitally missing teeth. The inclusion criteria were respected throughout the entire investigation. Among its limitations, this study was based on a convenience sample, since it is impossible to recruit the entire population of a big island. Also, it comprised two subgroups: an orthodontic and a non-orthodontic one, for the reasons explained above.

The observed prevalence of congenitally missing permanent teeth at 5.5% closely mirrors results from studies by Rakhshan and Rakhshan [30], Khalaf et al. [1], Bozga et al. [31], and Altug-Atac et al. [32]. Moreover, our findings of a female predilection align with previous research. Such findings were reported consistently by Muller et al. [33], Ioannidou-Marathiotou et al. [34], and Polder et al. [2]. A recent systematic review and meta-analysis conducted by Rakhshan and Rakhshan [30] reported a mean prevalence of 6.4% for congenitally missing teeth in males and 7.5% in females. Overall, the condition appeared to be more prevalent in females, with the trend observed primarily in epidemiological samples in the general population and not in orthodontic or dental patients. The inclusion of orthodontic or dental patients could possibly influence the observed rates, potentially increasing its occurrence in boys and/or decreasing it in girls. The observations of gender dimorphism remained unaffected by various factors, such as chronological or geographical variation. Moreover, no significant predominance of maxillary or mandibular involvement was noted, although the anterior segment was more likely to be affected [30]. Also, this meta-analysis pooled data from a number of studies with patients from various geographic regions; with Greece not being one of them. In fact, various ethnic groups encompassing Mongoloids, Negroids, Caucasians of European descent (encompassing Europeans, Americans, Australians, and Hispanics), and Caucasians from Asian nations made up the pooled sample. Based on a Mann–Whitney U test they performed, Europeans demonstrated a marginally significantly increased prevalence. Therefore, certainty on this matter may be questionable.

Regarding the distribution of missing teeth between the jaws, our findings on mandibular predilection contradict the aforementioned meta-analysis but resonate with the observations of Ioannidou-Marathiotou et al. [34] and Endo et al. [35]. The findings that the mandibular second premolars were the teeth whose absence was the most common comes in agreement with the observations by Ioannidou-Marathiotou et al. [34], Polder et al. [2], Bozga et al. [31], Khalaf et al. [1], and Badrov et al. (2017) [36]. Nevertheless, the order of prevalence of missing teeth does not align with all the patterns reported in the literature.

Also, although our study identified a higher prevalence of Angle’s Class II malocclusion, conflicting results exist in the existing literature. Gupta et al. reported no association between hypodontia and the various Angle’s malocclusion Classes in their research article [37].

The aforementioned study produced some additional results that did not totally agree with our findings [37]. Notably, they identified a tooth agenesis prevalence reaching 7.48%. The primary absent tooth was the maxillary lateral incisor (48.61%), followed by the mandibular lateral incisor (19.44%), the mandibular central incisor (8.33%), the mandibular second premolar (6.294%), and the maxillary second premolar (5.55%). Hypodontia manifested more frequently in the upper jaw. While Class I malocclusion patients exhibited the highest occurrence of hypodontia (7.87%), followed by Class II malocclusion patients (6.99%), it was least prevalent in Class III malocclusion patients. Despite these variations, there was no statistically significant difference in hypodontia across different malocclusion Classes. A reasonable explanation for the disparities among the results can be attributed to the nature of the samples. While our sample was based on White Europeans from a Greek island, Gupta and co-workers’ cohort were orthodontic patients from a university clinic based in Nepal. Therefore, it could be assumed that a significant part of the group was of South East Asian ancestry. It seems that the absence of different teeth can be affected by ethnicity, sample types (epidemiological or dental patients), sample sizes, and the minimum ages of the investigated subjects.

In this context, another study that was conducted by Costa et al. (2017) on a Brazilian population detected that the most prevalent skeletal malocclusion was Class I (63.11%), followed by Class II (25.94%), and Class III (10.95%) [38]. The average number of congenitally missing teeth per individual was 1.3. There was an equal number of subjects missing premolars and upper lateral incisors. Other teeth that were identified to be missing were lower incisors and molars. Finally, they found a negative correlation between the ANB angle and the number of congenitally missing teeth.

While congenitally missing teeth can be associated with various syndromes, non-syndromic and familial forms are more prevalent, as noted by Peker et al. [39]. Studies, such as the one by Sisman et al., suggest an increasing frequency of congenitally missing teeth in recent decades [40]. Modern individuals with fewer developed teeth exhibit smaller facial structures, with this effect becoming more pronounced as the number of missing teeth increases [41]. Craniofacial morphology and growth patterns in individuals with congenitally missing teeth are distinct, featuring a shorter maxilla, a more prognathic mandible, a smaller mandibular plane angle, and greater retroclination of the maxillary and mandibular incisors, as observed in studies by Ben-Bassat and Brin [42], Wisth et al. [28], Roald et al. [43], Sarnäs and Rune [44], Ogaard and Krogstad [45], and Endo et al. (2004) [35].

The initial clinical indication of a missing permanent tooth may manifest as the retention or submersion of a primary tooth; a clinical sign that can also be confirmed with radiographic examination. It should be noted that the initiation of calcification for the first premolars usually happens during 18–24 months of age and for the second premolars at 24–30 months of age [46]. Nevertheless, the absence of formation of a permanent tooth before the expected time should not always be mistaken for agenesis [47].

Regarding treatment, it is highly dependent on the etiology of congenitally missing permanent teeth. For example, in a case of ectodermal dysplasia, early intervention in the primary dentition is required. In instances where conical teeth are present within the oral cavity, a range of cosmetic interventions such as composite reshaping, composite resin crowns, composite veneers, traditional crowns, and meticulously crafted laboratory-fabricated bridges can be skillfully designed to enhance the overall aesthetic appeal. Subsequently, to uphold the vertical dimension, a partial denture—whether conventional or overdenture—can be constructed. It is imperative to acknowledge that the absence of alveolar bone development in the area of missing teeth may pose inherent challenges to denture retention. Nevertheless, adaptive measures, such as denture relining or comprehensive reconstruction, can be implemented to seamlessly accommodate the ongoing processes of growth and development, while ensuring optimal retention. An alternative method to sustain denture retention or secure removable appliance clasps involves the strategic use of composite resin or orthodontic buttons, strategically bonded to the existing teeth. Despite the conventional recommendation for implant placement post-craniofacial growth completion, the presence of ectodermal dysplasia may warrant the consideration of earlier implant placement [47]. In fact, the existing literature suggests a strategic approach to implant placement in the canine region of the mandible between the ages of 8 and 10 years. This strategic placement is performed to substantially enhance the retention of the lower denture, aligning with the culmination of the maximal transverse growth of the mandible [48].

In the scenario of mandibular incisor agenesis, it is worth noting that intervention for space closure may not be deemed necessary [47].

On the other hand, the initiation of coordinated efforts among specialized dentists is paramount to establishing a well-structured treatment plan for primary molars that lack permanent successors right from the moment of diagnosis. As a broad guideline, the recommendation leans towards retaining primary teeth without successors to effectively safeguard the alveolar ridge, offering support for future implant placement, and facilitating the integration of prosthetic crowns. The continual monitoring of bone levels between the submerged primary molar and its adjacent teeth through periapical radiographs is crucial, ensuring the preservation of horizontal bone levels without compromising vertical bone architecture. To optimize the space for eventual implant placement and the incorporation of prosthetic crowns, a strategic reduction in the mesiodistal width of a primary molar through disking is suggested, given that primary molars typically exhibit a width 1–2 mm greater than their successors [12], with the average size of a mandibular second premolar being 7.5 mm [49]. This reduction can be achieved by measuring the width of the primary molar at the cemento-enamel junction on a bitewing or periapical radiograph, or by comparing it to the contralateral premolar.

In cases where the primary molar is submerged, the application of composite resin to the occlusal surface is recommended to maintain a leveled occlusal plane and to prevent the overeruption of the antagonist. Optimal aesthetics, occlusal height, and the necessary occlusal width for future implant considerations can be achieved through the placement of a zirconia or composite resin crown [50]. Additional viable options include opting for a stainless-steel crown or extracting the primary molar post-eruption of the permanent one, coupled with space maintenance and/or orthodontic space closure. In situations where the treatment plan excludes orthodontic space closure, a space maintainer can effectively retain space until an implant is placed post the completion of facial growth. In instances where a permanent successor is absent, the retention of a primary molar in the oral cavity can be a viable long-term solution [50,51].

Various treatment options are available for addressing the absence of permanent lateral incisors. Removable appliances, canine substitution coupled with space closure, resin-bonded fixed partial dentures (FPDs), conventional full-coverage FPDs, and osseointegrated implants are among the alternative approaches. In situations where achieving satisfactory aesthetics is feasible, the primary lateral incisor can serve as a substitute for the permanent successor through orthodontic space closure.

In cases where mandibular second premolars are missing, an interdisciplinary treatment approach becomes imperative. The restorative treatment options can be broadly categorized into two groups: single-tooth implant and tooth-supported restoration. The tooth-supported restoration includes alternatives such as a resin-bonded FPDs, a cantilevered FPD, and a full coverage FPD. Collaboration between a dentist and an orthodontist is typically necessary to properly position the adjacent teeth in alignment with the proposed treatment plan [49]. Aesthetic improvements can be achieved through the use of a removable appliance featuring a prosthetic tooth as a transitional replacement before the completion of facial growth and prior to any orthodontic intervention. However, it is worth noting that a removable appliance is not the preferred retention method post-orthodontic treatment, as the convergence of central incisors and canines’ roots could complicate future implant placement. Conversely, fixed retention methods such as a bonded lingual wire with a prosthetic incisor, or a laboratory-fabricated resin-bonded bridge or porcelain-fused-to-metal bridge, are preferable until facial growth completion [49].

Following the completion of growth, the treatment of choice for a missing incisor involves a single-tooth implant. Ensuring the completion of growth is best achieved through the superimposition of a series of cephalometric radiographs at 6-month and 1-year intervals, as hand–wrist radiographs may not accurately predict the cessation of facial growth. Implant placement is typically performed around 20–21 years of age in males and 16–17 years of age in females [49].

Patient preference also plays a role in the decision of the definitive treatment since undergoing surgery and/or ridge augmentation or orthodontic treatment may not be a choice. The resin-bonded bridge is favored as the least invasive option for tooth replacement, although it is accompanied by the potential issue of debonding. Hence, meticulous patient selection is imperative to ensure the longevity of a resin-bonded bridge, particularly favoring cases with a shallow overbite, nonmobile upright abutments, and an absence of bruxism. Conversely, the cantilevered FPD offers increased predictability when all contacts in excursive movements are eliminated. The utilization of a full-coverage FPD is advisable only when replacing an existing full-coverage bridge or addressing caries or fractures in adjacent teeth, given its more invasive nature [47].

Alternatively, canine substitution presents itself as a viable option for managing the absence of maxillary lateral incisors, requiring collaboration between a general dentist or prosthodontist and an orthodontist. The process involves achieving space closure through orthodontic tooth movement, relocating the canine into the lateral incisor’s space, and subsequently modifying the canine’s shape to resemble a lateral incisor through enameloplasty. Challenges may arise regarding the canine’s size, color, root volume, and the height of the gingival margin. Post-reshaping, bleaching, or restoration with veneers or composite for aesthetic purposes, the adjacent premolar may end up shorter and narrower than the contralateral canine, necessitating additional intervention with veneers or potentially extraction [52]. A comparative analysis of prosthetic treatment versus canine substitution revealed that orthodontic space closure was superior in terms of functional, periodontal, and esthetic outcomes [53]. However, the choice between space closure and canine substitution is not always straightforward, as the treatment plan must carefully consider numerous factors.

Despite the existing research dedicated to understanding the prevalence of congenitally missing permanent teeth, there remains a noticeable scarcity of case–control or cohort studies that thoroughly examine the associated risk factors. Another area ripe for exploration involves conducting additional genetic studies to elucidate potential associations between congenitally missing teeth and specific genes. An intriguing avenue for future researchers lies in delving into the prevalence of congenitally missing teeth within twin pairs or across family trees spanning generations. However, it is important to acknowledge the inherent challenges in acquiring suitable samples for such investigations.

Congenitally missing permanent teeth can have various impacts on the stomatognathic system, influencing the position of adjacent and opposing teeth, modifying occlusal function and relationships, and leading to aesthetic concerns and asymmetry [45]. Addressing this condition may necessitate an interdisciplinary approach with a collaboration among the fields of oral surgery, operative dentistry, orthodontics, and prosthodontics [54].

Given the considerable prevalence of congenitally missing permanent teeth in the literature and in our study [15,32,36,55], coupled with considerations of both aesthetic and functional implications [2,27,39,56,57,58,59,60,61,62], and the necessity for complex and costly treatments [63], the clinical problem of congenitally missing permanent teeth is of significant importance [32].

## 5. Conclusions

The prevalence of congenitally missing teeth was 5.5% and 4% in the orthodontic and non-orthodontic groups, respectively, with the lower second premolar being the most commonly absent, followed by maxillary lateral incisors. Females showed a predilection in both orthodontic and non-orthodontic samples. The mandible had the highest number of missing teeth in the orthodontic sample.

## Figures and Tables

**Table 1:** Distribution of individuals with congenitally missing teeth [n of children (%)].

	Orthodontic Group [521 Children]	Non-Orthodontic Group [100 Children]
Males	14 (2.7%)	1 (1%)
Females	15 (2.8%)	3 (3%)
Total	29 (5.5%)	4 (4%)

**Table 2:** Distribution of congenitally missing teeth by tooth type [n of teeth] in the orthodontic sample and non-orthodontic samples.

	Orthodontic Sample														Non-Orthodontic Sample													
	17	16	15	14	13	12	11	21	22	23	24	25	26	27	17	16	15	14	13	12	11	21	22	23	24	25	26	27
Males						2			1			1																
Females			1			6			3											2			1					
	47	46	45	44	43	42	41	31	32	33	34	35	36	37	47	46	45	44	43	42	41	31	32	33	34	35	36	37
Males			3		2		4	3				5														1		
Females	1		4									8		1			1											

**Table 3:** Distribution of individuals with congenitally missing teeth per Angle’s Class in the orthodontic group sample [n of children (%)].

	Class I	Class II	Class III
Males	3 (10%)	10 (34%)	1 (4%)
Females	7 (24%)	8 (28%)	
Total	10 (34%)	18 (62%)	1 (4%)

**Table 4:** Distribution of congenitally missing teeth by tooth type [n of teeth] across Angle’s Classes.

	Class I														Class II													
	17	16	15	14	13	12	11	21	22	23	24	25	26	27	17	16	15	14	13	12	11	21	22	23	24	25	26	27
Males												1								2			1					
Females			1			2			1											4			2					
	47	46	45	44	43	42	41	31	32	33	34	35	36	37	47	46	45	44	43	42	41	31	32	33	34	35	36	37
Males			1		1							2					1		1			3				3		
Females												5			1		3				4					3		1

## Data Availability

The supporting data reported can be provided upon request to the corresponding author.
